# Serum glial fibrillary protein reflects early brain injury dynamics and cognitive changes after deep brain stimulation surgery

**DOI:** 10.1038/s41598-025-00399-3

**Published:** 2025-05-13

**Authors:** Anika Frank, Jonas Arjomand, Jonas Bendig, Mia Delfs, Lisa Klingelhoefer, Witold H. Polanski, Katja Akgün, Tjalf Ziemssen, Björn Falkenburger, Nils Schnalke

**Affiliations:** 1 Department of Neurology, University Hospital Carl Gustav Carus Dresden, Fetscherstr. 74, 01307 Dresden, Germany; 2 Center for Neurodegenerative Diseases within the Helmholtz Association (DZNE), Dresden, Germany; 3 Department of Neurosurgery, University Hospital Carl Gustav Carus Dresden, Dresden, Germany; 4 Department of Neurology, Klinik am Tharandter Wald, Halsbrücke, Germany

**Keywords:** Parkinson’s Disease, Dystonia, Tremor, DBS, sNfL, sGFAP, Movement disorders, Parkinson's disease

## Abstract

Deep brain stimulation (DBS) is an efficient treatment for movement disorders, most commonly Parkinson’s Disease (PD), dystonia and essential tremor. DBS surgery carries risks, e.g. the risk of delayed peri-lead edema (PLE) and the risk of postoperative cognitive decline. The mechanisms of these complications are not fully understood and there is no established biomarker to screen for these complications after DBS surgery. To explore the diagnostic value of two blood-based markers representative for distinct types of brain injury, we characterized the dynamics of serum glial fibrillary acidic protein (sGFAP, for glial injury) and serum neurofilament light chain (sNfL, for neuronal-axonal injury) following DBS surgery. We analyzed longitudinal dynamics of serum protein levels in 58 patients undergoing deep brain stimulation (DBS) at our center for half a year post-surgery. Serum GFAP responded much more rapidly after brain surgery, returning to baseline after weeks, whereas sNfL only returned to baseline after months. Patients with lower preoperative cognitive performance exhibited higher postoperative sGFAP levels, with sGFAP showing a stronger association with preoperative patient characteristics compared to sNfL. Further studies with long-term clinical follow-up are needed to fully evaluate the utility of sGFAP as a biomarker for both early and delayed complications after DBS surgery, including cognitive decline and potential foreign body reactions to the implanted lead.

## Introduction

Deep brain stimulation (DBS) of target nuclei such as Globus pallidus pars interna (GPi), Nucleus subthalamicus, (STN) and thalamus (ventral intermediate, VIM) is an established and efficient treatment for movement disorders. Most commonly, DBS is considered in essential tremor (ET), dystonia and Parkinson’s disease (PD)^1–4^. In general, DBS is safe and effective also in older patients and is still considered in patients with cognitive impairment^5,6^.

DBS surgery carries risks, and some of them are known to be influenced by preoperative patient characteristics. While DBS for People with Parkinson’ disease (PwPD) is generally considered safe in regards of cognitive side effects, there are some studies raising concern about postoperative cognitive decline, especially when targeting the STN^7,8^. This risk increases with age^9^, preoperative cognitive impairment^10–12^ or genetic risk factors^13^. The mechanism for this is still unknown and thus, it is currently not possible to accurately predict postoperative cognitive decline, and further research is needed to clarify the long-term cognitive outcomes following DBS.

Another risk associated with DBS surgery is brain hemorrhage, which has been significantly reduced through advancements in imaging and surgical techniques as trajectories can avoid blood vessels and fewer microelectrode tracks are necessary for accurate lead placement^14^. However, delayed-onset peri-lead edema (PLE) is still observed after DBS surgery with one study reporting symptomatic PLE in 7% (out of 14.7% total prevalence in postoperative MRI) of patients 6 weeks after DBS surgery. Symptoms included confusion, seizures and transient focal neurological deficits like aphasia and hemiparesis^15^. Additionally, postoperative brain edema has been linked with cognitive decline and might therefore be and additional risk factor for short and long-term cognitive deterioration after lead implantation^16^. The pathophysiology of PLE is not fully understood, and it is commonly attributed to a foreign body reaction^17^. Still, other mechanisms are conceivable, e.g. the implantation of DBS electrodes could be akin to processes described in traumatic brain injury (TBI), in which a role of the response of glial cells to the injury in addition to neuronal damage is assumed^18^.

In a previous study, we showed that neuronal injury as reported by serum neurofilament (sNfL) is caused by DBS surgery itself and not by chronic neurostimulation^19^. Unfortunately, the half-life of sNfL is long, so sNfL increases slowly and only returns to baseline values months after surgery. Serum NfL is therefore not an ideal marker to assess acute perioperative damage in DBS surgery.

As serum glial fibrillary acidic protein (sGFAP) is a commonly used and reliable biomarker for glial injury, which has been approved for clinical use in traumatic brain injury (TBI) by the FDA due to its favorable relation to clinical outcomes and its kinetics after injury^20^, it might offer a useful alternative. Studies in Multiple Sclerosis (MS) suggest a better correlation of sGFAP values with factors of disease progression than for sNfL^21,22^. In PD, sGFAP elevations are potentially linked to disease progression^23^, cognitive decline^24^, and specific motor subtypes (e.g., postural instability and gait difficulty (PIGD)^25,26^. Moreover, sGFAP could potentially be useful to unravel the role of glial injury in the pathogenesis of PLE or help to identify patients at risk for postoperative cognitive decline.

To examine whether sGFAP might constitute a suitable marker of peri- and postoperative damage of DBS surgery, we measured sGFAP along with sNfL pre- and postoperatively in DBS patients receiving either GPi, VIM or STN-DBS. In addition, we assessed whether preoperative patient characteristics (related to the neurodegenerative nature of the disease as well as metabolic risk profiles), surgery modalities (asleep/awake and insertion of microelectrodes) or biomarker profiles are associated with an increased the risk of neuronal or glial injury as reported by sNfL and sGFAP.

## Results

### Serum neurofilament after DBS surgery

A total of 58 patients undergoing DBS surgery in our center were prospectively enrolled in this study, comprising 47 patients with PD and 11 patients with “non-degenerative” diseases, i.e., dystonia or ET. Their demographic characteristics are displayed in Table 1**.**

**Table 1:** Demographic and clinical characteristics.

	PwPD (n = 47)	Non-degenerative (n = 11)
Age at DBS implantation, years	63.3 (8.8)	59.3 (15.9)
Sex, n male/female (% female)	31/16 (34%)	8/3 (27%)
Disease duration, years	9.5 (3.9)	27.9 (23.1)
Hoehn and Yahr stage, median (range)	2 (1–4)	n/a
UPDRS III OFF total score	39.6 (16.3)	n/a
Motor phenotype		n/a
Tremor-dominant, n (%)	13 (27.7%)	
Akinetic-rigid, n (%)	15 (31.9%)	
Mixed, n (%)	19 (40.4%)	
MoCA total score	26.8 (2.5)	n/a
preoperative sNfL, pg/ml	18.8 (7.4)	17.9 (7.4)
preoperative sGFAP, pg/ml	139.8 (71.7)	153.5 (97.1)
Postoperative complications		
delirium, n (%)	4 (8.5%)	0 (0%)
hemorrhage of any kind, n (%)	2 (4.3%)	0 (0%)

Data are “mean (SD)” unless otherwise indicated. *UPDRS* Unified Parkinson’s Disease Rating Scale; *MoCA* Montreal Cognitive Assessment, *sNfL* serum neurofilament light, *sGFAP* serum glial fibrillary acidic protein.

**Fig. 1:**
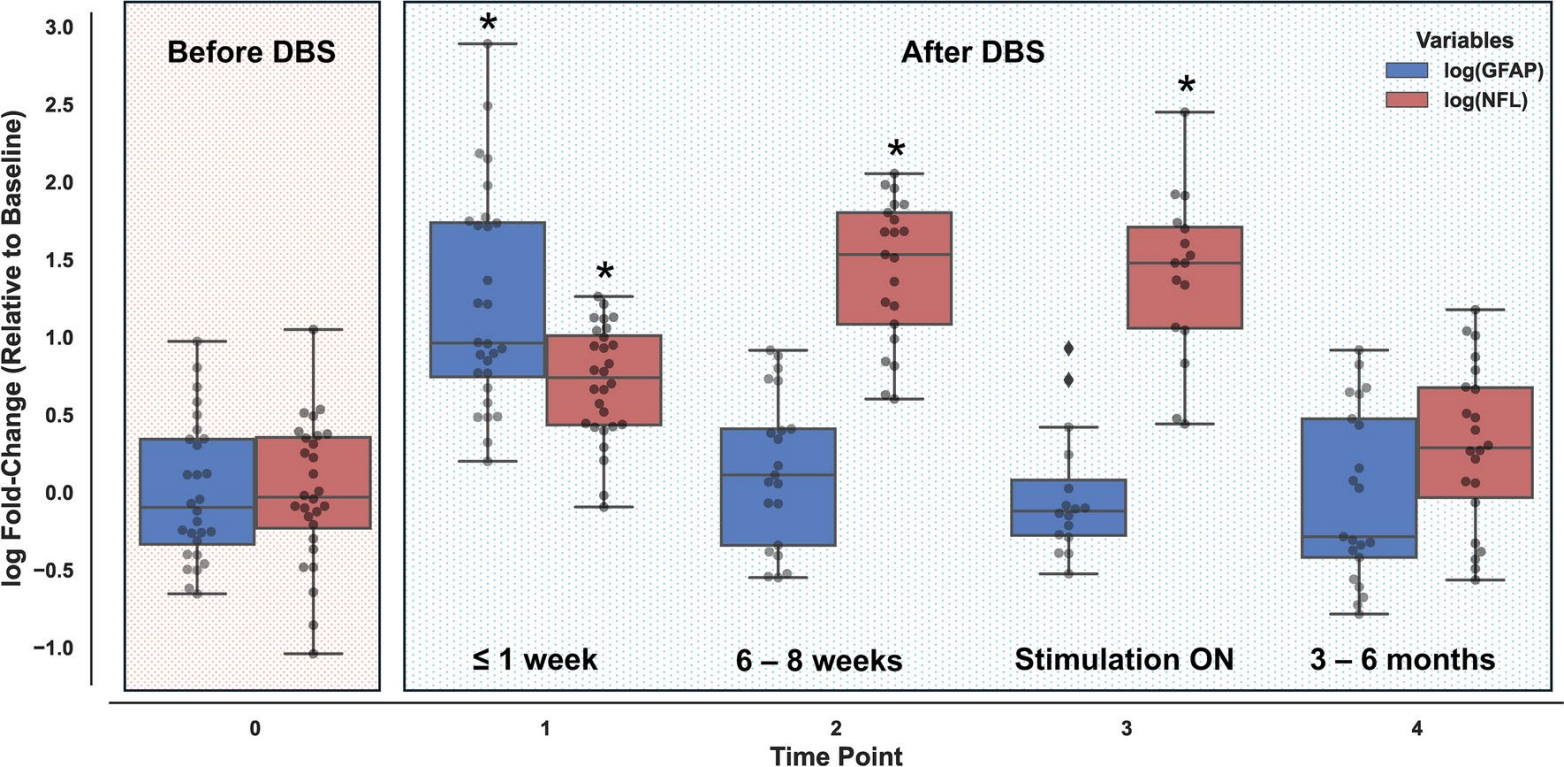
Log fold-change of log(sNFL) and log(sGFAP) measurements after STN-DBS in PwPD. Values are set in relation to baseline log values. Asterisks indicate values significantly different from baseline with p ≤ 0.001, all other comparisons to baseline values: ns. Mean log(sGFAP) values: blue, mean log(sNfL) values: red.

To report neuronal injury during and after DBS surgery, sNfL was used as previously described^19^. In patients with PD, sNfL increased significantly (Friedman’s test and Nemenyi-Friedman post-hoc test, p < 0.001) between preoperative baseline and one day after surgery (Fig. 1). The mean preoperative baseline sNfL was 18.8 pg/ml (95% CI 16.24–21.36), sNfL increased to 40.92 pg/ml (33.071–48.78) at the first postoperative time point. At the second postoperative time point, mean sNfL was 79.88 pg/ml (68.72–91.043) and significantly higher (p < 0.001) than preoperative baseline or the first and fourth postoperative time points. There was a tendency of sNfL to decline at the third postoperative time point (mean sNfL 75.64 pg/ml (62.67–88.61), p = 0.755 for comparison with time point 2). The fourth postoperative measurement was obtained 3–6 months after surgery, mean sNfL was 23.34 pg/ml (19.21–27.46), which was not significantly different from preoperative baseline (p = 0.619), thus indicating a return to baseline values. In patients with non-degenerative diseases, the results were equivalent. Due to the small sample size, the preoperative baseline vs. the first postoperative time point was not significantly different, the other results described above also apply to this part of our cohort.

A two-way ANOVA between the PD and the non-degenerative group was significant for the interaction between the time points after surgery and log(sNfL) measurements in both groups (p < 0.001), i.e. it showed an effect of surgery on log(sNfL) values. There was no significant effect of either the group itself or the interaction between the group and the time point on log(sNfL) measurements.

Taken together, the time course of sNfL after DBS surgery in patients with PD was similar as described previously^19^, findings in patients with essential tremor or dystonia were similar to those observed in PwPD.

### Serum GFAP after DBS surgery

The slow time course of sNfL after surgery results mainly from its long half-life and not from continual neuronal damage – as discussed previously^19^. To obtain more direct information about damage resulting from DBS surgery, we measured sGFAP. Since GFAP is expressed in astrocytes, it reports glial damage, which is another difference to sNfL. The time course of sGFAP was markedly different from sNfL in PD patients (Fig. 1).

In PwPD, only the first postoperative time point differed significantly from preoperative baseline (p = 0.001) and subsequent time points (p for all pairwise comparisons ≤ 0.005). Mean sGFAP was 136.65 pg/ml (113.51–159.78) at preoperative baseline, 540.06 pg/ml (361.5–718.62) at the first postoperative time point, 166.56 pg/ml (134.27–198.85) at the second postoperative time point, 150.35 pg/ml (115.26–185.44) at the third postoperative time point and 145.43 pg/ml (111.37–179.49) at the fourth postoperative time point. Postoperative time points 2–4 were not significantly different from preoperative baseline. The time course for the non-degenerative cohort was similar in that the first postoperative measurement was the only one that differed significantly from baseline.

Again, a two-way ANOVA revealed no significant interaction between the group (i.e. PD and non-degenerative) and postoperative log(sGFAP) measurements and a significant effect of the time point after surgery and log(sGFAP) measurements (p < 0.001).

Note that log(sNfL) and log(sGFAP) values for PwPD are displayed in Fig. 1 in relation to baseline values to enable comparability between serum marker kinetics. The results of post-hoc tests comparing measurements between time points were identical when using log(sNfL) and log(sGFAP) instead of raw sNfL or raw sGFAP.

### Baseline values of sNfL and sGFAP are affected by age and BMI, but not disease specific factors in PwPD

The correlation between log(sNfL) and log(sGFAP) of the same patient at preoperative baseline was moderate in PwPD (Spearman’s rho, r = 0.55, p = 0.002 in PwPD; r = 0.45, ns in non-degenerative patients). This could result from a floor effect or from the fact that sNfL reports neuronal damage and sGFAP reports glial damage. It is consistent with the observation that the time course of the two parameters and the factors that influence them are different^27–29^.

**Fig. 2:**
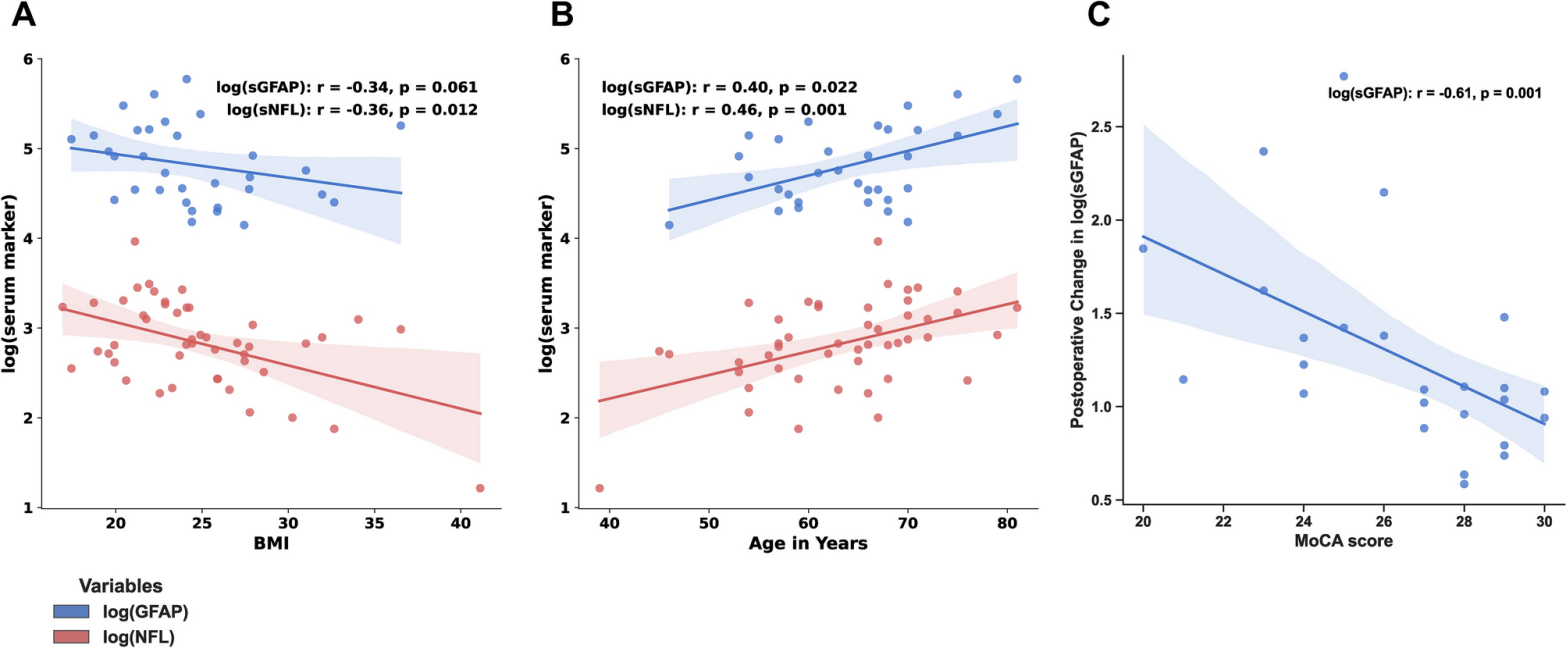
Correlations of clinical variables with markers of neuronal injury in PwPD after DBS. (**A**) BMI; (**B**) Age, (**C**: MoCA Score. Note that panel (**C**) shows the correlations of preoperative MoCA scores with the first postoperative measurement of log(sGFAP), the other panels show correlations with baseline serum marker measurements. Log(sGFAP): blue, log(sNfL): red.

Baseline values of sNfL and sGFAP are influenced by factors that may increase their release into the blood, including age and the presence of neurodegenerative diseases, and by factors that may affect their distribution or elimination, like body mass index (BMI) or renal function^22,30,31^. Indeed, baseline log(sNfL) correlated moderately negatively with BMI (Fig. 2a; PwPD: r = − 0.36, p = 0.012; non-degenerative patients: ns). Baseline log(sGFAP) did not correlate significantly with BMI in PwPD (r = − 0.34, p = 0.061), but in non-degenerative patients (r = − 0.7, p = 0.016). In PwPD, baseline log(sNfL) and log(sGFAP) values also correlated with age (Fig. 2b**,** log(sNfL) in PwPD: r = 0.46, p = 0.001, log(sNfL) in non-degenerative patients r = 0.69, p = 0.018; log(sGFAP) in PwPD r = 0.4, p = 0.022, log(sGFAP) in non-degenerative patients: ns).

There was a significant correlation between MoCA scores and baseline log(sNfL) values (r = − 0.32, p = 0.043), as has been described previously^32,33^. There was no significant correlation between MoCA scores and baseline log(sGFAP). Baseline log(sGFAP) and log(sNfL) did not correlate significantly with further PD-specific assessments (UPDRS III total score, Hoehn and Yahr-stage, presence of dyskinesias or other disease related complications, not shown). Also, baseline log(sGFAP) and log(sNfL) did not correlate significantly with comorbidities, such as diabetes, arterial hypertension, uremia and nicotine abuse (Spearman’s rho), independent of group (PwPD vs. non-degenerative patients, Mann–Whitney-U).

### Perioperative damage as reported by sGFAP is influenced by age, motor and cognitive symptoms and baseline sGFAP

We observed a strong correlation between values at baseline and the first postoperative time point in PwPD, both for log(sNfL) (log(sNfL) r = 0.71, p < 0.001) and for log(sGFAP) (r = 0.72, p < 0.001). These correlations were also observed in non-degenerative patients (only significant for log(sNfL) r = 0.8, p = 0.003, log(sGFAP): ns). Patients with higher values at baseline thus showed higher postoperative values of log(sNfL) and log(sGFAP).

To identify risk factors of increased perioperative damage, we aimed to predict postoperative log(sGFAP) and log(sNfL) values in PwPD from baseline data by using a random forest regressor. A random forest regressor predicted postoperative values at chance level. Using gradient boosting, however, a model incorporating age, UPDRS III in the OFF condition, the total MoCA score and the baseline log(sGFAP) value explained about 50% of the variance in postoperative log(sGFAP) values (r^2^ = 0.49). Of the factors in this model, the relative importance of preoperative log(sGFAP) was by far the highest (0.7), with all other factors having a combined importance < 0.3. This could not be replicated for log(sNfL) values, indicating that GFAP could be better suited to report differences in perioperative damage between individual patients.

To explore these findings in more detail, we examined individual correlations with log(sNfL) and log(sGFAP) values. In contrast to the findings with gradient boosting, the degree of change of log(sNfL) and log(sGFAP) did not correlate significantly with age. MoCA total values were negatively correlated to the degree of change in log(sGFAP) values with r = -0.61 (Fig. 2c, p = 0.001), i.e., patients with cognitive impairment showed more perioperative damage as reported by log(sGFAP). The GPi is often used as a DBS target in patients with cognitive impairment. To ensure that the correlation of log(sGFAP) and MoCA was not biased by this, we computed the correlation without PwPD that received GPi-DBS. The correlation remained moderate with r = − 0.54 (p = 0.008). The correlation of MoCA with log(sNfL) was not significant. Correlations of baseline log(sNfL) and log(sGFAP) with the maximum change from baseline (second postoperative time point for log(sNfL) and first postoperative time point for log(sGFAP)) were also not significant.

The change in log(sGFAP) or log(sNfL) after surgerydid not correlate significantly with further PD-specific assessments (UPDRS III total score, Hoehn and Yahr-stage, presence of dyskinesias or other disease related complications, not shown), or with comorbidities (diabetes, arterial hypertension, uremia, nicotine abuse). BMI was moderately positively correlated to the degree of change of log(sNfL) in PwPD (r = 0.5, p = 0.02), but not in non-degenerative conditions. There was no significant correlation with the degree of change of log(sGFAP). This correlation was reversed, but not significant when controlling for preoperative log(sNfL), indicating that this correlation is driven by baseline values (partial correlation r = − 0.59, p = 0.07). The observation itself might be explained by an increased distribution volume in patients with a higher BMI.

We used a mixed-linear model to test if log(sNFL) and log(sGFAP) dynamics following DBS surgery differ across motor phenotypes (tremor-dominant, akinetic-rigid and mixed, Suppl. Fig. 1). The main effects for motor phenotypes (i.e. global effect of motor phenotype across all time points) were not significant for both markers. Nevertheless, we found stronger increases of log(sGFAP) at the first postoperative time point in akinetic-rigid compared to tremor-dominant type (p = 0.034 for interaction effect). We observed a similar relationship in log(sNFL), which showed a significantly stronger increase at the third postoperative time point in mixed compared to tremor-dominant type and the same trend for the akinetic-rigid type (p = 0.030 and p = 0.059 for interaction effect, respectively).

Factors of the DBS surgery itself, i.e., the duration of the operation, the number of microelectrodes used, or asleep/awake surgery and the total energy delivered by the stimulation at different time points, did not show any significant association (Spearman’s rho) or significant differences (Mann–Whitney-U) between groups. However, the change in log(sGFAP) after surgery was significantly higher in patients with immediate post-operative complications (p = 0.014, Mann–Whitney-U, n = 6 in total, see Table 1), while we did not observe any differences in the change in postoperative change in log(sNFL) (p = 0.805, Mann–Whitney-U).

## Discussion

In this study, we performed longitudinal measurements of serum biomarkers of neuronal (sNfL) and glial (sGFAP) injury after DBS surgery in a cohort of patients with movement disorders, namely PD, dystonia and ET. To the best of our knowledge, this is the first study reporting prospective postoperative measurements of sGFAP in DBS patients. Both sNfL and sGFAP exhibited a temporary increase following surgery, but sGFAP displayed a significantly faster kinetic response compared to sNfL.

We found no relevant differences in serum marker measurements between PD and non-degenerative disorders, i.e., dystonia and ET or metabolic predisposition (i.e., presence of arterial hypertension, diabetes, or uremia). Moreover, neither asleep versus awake surgery nor the number of microelectrodes inserted influenced postoperative biomarker levels. While a previous study suggested a near-significant trend toward increased neuro-axonal damage with a greater number of microelectrode recordings, this association was not observed for sGFAP^19^. We therefore hypothesize that the postoperative sGFAP increase results from a foreign body reaction^17^ rather than direct intraoperative trauma, which would be more severe with a higher number of microelectrode tracts. It should be noted, however, that the variance of microelectrode tracks was small and that the first post-operative time point was 3–5 days after the DBS implantation, potentially missing an initial sGFAP peak caused by the insertion of microelectrodes^34^. Furthermore, the sensitivity of sGFAP might not be sufficient to detect small differences in traumata induced by thinner microelectrodes compared to DBS leads with a higher diameter. Since the perioperative change in sGFAP and baseline sGFAP were moderately correlated, non-injury-associated factors like elimination mechanisms observed in sNfL could play a role in the observed changes.

Baseline values of sNfL where higher in older participants (Fig. 2b), consistent with previous findings by others^35^. We also observed a negative association between sNfL and BMI (Fig. 2a), which has been explained by the increased distribution volume of NfL released from the nervous system^36^. Apart from a non-significant correlation between BMI and sGFAP in PwPD, these observations also apply to sGFAP (Fig. 2a and b), as has been observed in MS^22,30^. In contrast to Che et al.^25^ we did not find a direct association between motor phenotype and sGFAP, however, we were able to identify different trajectories in postoperative sGFAP and sNfL dynamics with stronger biomarker increases in mixed or akinetic-rigid types compared to the tremor-dominant type. This relationship could point to a higher vulnerability in selected motor phenotypes and warrants further investigation.

Serum GFAP transiently increased after DBS surgery, returning to baseline values just weeks after surgery (Fig. 1). This is markedly different from sNfL, which only returned to baseline 3 – 6 months after surgery (Fig. 1 and previous reports^19^). Elevated levels of sGFAP^24^ and sNfL^37^ are strongly associated with clinical milestones of PD (dementia, falls, nursing home placement and hallucinations) and with limited survival^38,39^. The fact that both biomarkers returned to baseline confirms the long-term safety of DBS therapy – albeit in patients carefully selected for this procedure. In particular, the lack of an increase of sGFAP or sNfL after initiation of electrical stimulation and the lack of an association of both biomarkers to the total energy delivered by DBS demonstrates that the stimulation is not harmful and does not by itself confer a risk of accelerated neurodegeneration^19^. Due to the rapid postoperative normalization, this finding is clearest for sGFAP (Fig. 1); the time course of sNfL confirms our previous findings^19^.

We hypothesized that the faster kinetic of sGFAP, compared to sNfL, might aid in detecting delayed injury after DBS surgery. Considering immediate postoperative complications (delirium n = 4, intracranial hemorrhage n = 2), we observed a significantly greater increase in the postoperative log(sGFAP) while no differences were found in log(sNfL). This is supportive of our hypothesis that sNfL is less influenced by certain immediate postoperative events. Other DBS-related complications in PwPD, such as peri-lead edema (PLE) and cognitive deterioration, may occur with a delay and hence could go undetected in routine post-surgical clinical monitoring.

PLE constitutes a rather commonly observed adverse event after DBS surgery, with at least 14.7% of DBS patients developing PLE after surgery, only half of which are symptomatic, i.e. presenting seizures or transient focal neurological deficits^15^. One study even reported some degree of PLE in all of 18 consecutively investigated DBS patients postoperatively^40^ and thus, PLE may be underdiagnosed in clinical practice. As discussed above, the pathophysiology of PLE is not fully understood but glial reactivity might contribute to the development of PLE, as has been shown for other forms of brain edema^41^. PLE typically occurs with a delay of up to 3 weeks after surgery^40^, hence at a time point when sGFAP has already returned to baseline in most cases (Fig. 1). Increasing or persistently increased sGFAP values during this time could therefore indicate additional injury. Although PLE tends to be a transient self-limiting phenomenon in most cases, more recent reports linked DBS-related postoperative brain edema to long-term cognitive deterioration^16^.

Glial reactivity^24^ and inflammatory responses^42^ are known to be heightened in PwPD with lower cognitive performance, which is supported by our finding of higher postoperative sGFAP values in this group. This might suggest an increased risk of PLE in this population, and in turn could further contribute to accelerated cognitive decline in this population. Even within the narrow range of MoCA scores in our cohort, a lower preoperative MoCA score was associated with a greater perioperative increase in sGFAP (Fig. 2c). This finding suggests that even moderate cognitive impairment heightens the risk for perioperative damage, supporting the practice of excluding patients with significant cognitive impairment from DBS surgery^43^. Nevertheless, our findings indicate that there is no clear threshold for a “safe” DBS surgery. Instead, safety seams to decline gradually with cognitive performance, thus constituting an argument for “early” DBS surgery in patients that are expected to benefit from the procedure. Additionally, our findings underline the importance of standardized preoperative cognitive testing in PD^44^.

There are limitations to this study. First, this was a single center study, and the results should be confirmed in a multicenter study. Second, a substantial number of measurements at the third time point are missing (35%) due to organizational aspects; the first activation of DBS in a few PwPD and in most patients with non-degenerative diseases was done in an outpatient setting. Third, the number of non-degenerative patients included in our cohort was low. These patients mainly represent a control group in comparison to PwPD, so our study was not able to identify possible risk factors for surgery unique to either dystonia or ET. While we were able to characterize the longitudinal kinetics of sGFAP versus sNfL after DBS surgery, we can only suggest sGFAP as a suitable biomarker for delayed postoperative complications involving glial response (i.e., PLE and delayed cognitive decline). This study did not test for the presence of PLE via brain imaging, nor did we conduct long-term follow-up on cognitive function. Therefore, additional studies are needed.

## Conclusion

In summary, our study explored different serum dynamics of markers of neuronal versus glial brain injury following DBS surgery. Due to its long half-life, sNfL measurements are less useful to detect additional or delayed brain injury after DBS surgery, such as PLE, because – as shown here and elsewhere^45,46^ – sNfL remains elevated for months after neuronal injury. Serum GFAP rises and falls more rapidly after surgery in comparison to sNfL. Therefore, sGFAP could serve as a blood-based biomarker for safety assessments, particularly when imaging should be avoided, is not available or inconclusive. Additionally, we observed a negative correlation between preoperative cognitive function, as measured by MoCA, and sGFAP, which was not seen for sNfL. This suggests that sGFAP is more consistently associated with preoperative risk factors and may be better suited for detecting perioperative damage and risk. As sGFAP (i) shows a favorable kinetic to detect acute injury after DBS surgery, (ii) its postoperative increase is associated with preoperative cognitive performance and (iii) indicates glial damage, it might be suitable to examine delayed post-DBS complications more systematically.

## Methods

### Study population and design

Patients were recruited at the University Hospital Dresden between March 2018 and November 2023. The study was approved by the institutional review board of the Technische Universität Dresden (EK533122019, EK 487122016, IRB 00001473). Written informed consent was obtained from all participants before inclusion in the study. All study procedures were performed in accordance with relevant local guidelines and regulations.

Measurements of serum markers were collected as follows: (i) *preoperative baseline (time point 0)*: within 2 days before surgery (n = 58), (ii) *postoperative time point 1*: 3–5 days postoperatively (n = 50), (iii) *postoperative time point 2:* 6–8 weeks after DBS surgery, before activation of stimulation (n = 49), (iv) *postoperative time point 3:* up to one week after activation of stimulation (n = 38) and (v) *postoperative time point 4:* 3–6 months after activation of stimulation (n = 51).

Demographic data were collected from all participants. For PD patients, this included further clinical information about motor and cognitive status (Hoehn & Yahr stage, Unified Parkinson’s Disease Rating Scale (UPDRS) part III in the OFF and ON condition, Montreal Cognitive Assessment (MoCA)) and the presence of disease-related complications. For all patients, we obtained information about common cardiovascular risk factors such as arterial hypertension, diabetes, uremia, BMI and smoking status.

A total of 64 patients were recruited for this study. The data of 6 patients were excluded from the analysis (all of them with PD), since either the baseline measurement was missing or less than half (i.e. 1 or 2) measurements had been performed. Thus, data of 58 patients were included in the analysis. Of the remaining 47 PwPD in our study, 44 received STN-DBS and 3 received GPi-DBS. All 3 dystonia patients received GPi-DBS and all 8 ET patients received VIM-DBS. Patients received implants of the following manufacturers: 45 Boston Scientific, 6 Medtronic, 4 Abbott and 3 Aleva Neurotherapeutics.

### sNfL and GFAP measurements

After preparation, serum samples were stored at − 20 °C. NfL measurement was performed as described previously, using the Advantage NF-Light Singleplex-Kit on a Simoa HD-1 instrument (Quanterix). Calibrators and diluted serum samples were measured in duplicate. The cut-off for variation of duplicates for the mean intraassay coefficient as well as the mean interassay coefficient was < 10%. Serum GFAP measurement was performed using the GFAP Singleplex-Kit from Quanterix on the same HD-1 instrument.

### Statistical analyses

Comparisons between groups were carried out using the Mann–Whitney-U-Test, adjusted with Bonferroni correction where applicable. To assess differences between different time points, a Friedman’s test was performed. To assess differences between different time points, a Nemenyi-Friedman post-hoc test was used, which inherently corrects for multiplicity. We used a two-way ANOVA to determine the influence of the group (i.e. PD or non-degenerative) and the time points after surgery on sNfL and sGFAP measurements.

To normalize the right-skewed distribution of NFL in further analysis, natural log-transformation (log(sNfL)) was used, as described by others. The same transformation was applied to serum GFAP measurements. Bivariate correlations were assessed with Spearman’s rank test. Since there is no global null-hypothesis assuming all correlations in the dataset are r = 0, these do not need to be corrected for multiplicity^47^.

To assess whether baseline data could predict postoperative log(sNfL) and log(sGFAP) values, a random forest regressor and gradient boosting were used. To test, whether log(sNFL) and log(sGFAP) dynamics following DBS surgery differ across motor phenotype, we used a mixed-linear model.

Statistical analyses were performed with Python. Sample size was not determined by a separate sample size estimate, as we tried to include all patients receiving DBS at our center from 2018 and 2023 that were available for assessments postoperatively and agreed to be included in this study.

## Supplementary Information

Supplementary Information.

## Data Availability

The data collected in this study are available from the corresponding author upon reasonable request.

## References

[1] Limousin, P. & Foltynie, T. Long-term outcomes of deep brain stimulation in Parkinson disease. *Nat. Rev. Neurol.* **15**, 234–242 (2019). 30778210 10.1038/s41582-019-0145-9

[2] Flora, E. D., Perera, C. L., Cameron, A. L. & Maddern, G. J. Deep brain stimulation for essential tremor: A systematic review. *Mov. Disord. Off. J. Mov. Disord. Soc.* **25**, 1550–1559 (2010). 10.1002/mds.23195 20623768

[3] Volkmann, J. et al. Pallidal neurostimulation in patients with medication-refractory cervical dystonia: A randomised, sham-controlled trial. *Lancet Neurol.* **13**, 875–884 (2014). 25127231 10.1016/S1474-4422(14)70143-7

[4] Tisch, S. & Kumar, K. R. Pallidal deep brain stimulation for monogenic dystonia: The effect of gene on outcome. *Front. Neurol.* **11**, 630391 (2021). 33488508 10.3389/fneur.2020.630391 PMC7820073

[5] Azevedo, P., Aquino, C. C. & Fasano, A. Surgical management of Parkinson’s disease in the elderly. *Mov. Disord. Clin. Pract.* **8**, 500–509 (2021). 33981782 10.1002/mdc3.13161 PMC8088115

[6] Al Ali, J. et al. Cognitive outcomes in patients with essential tremor treated with deep brain stimulation: A systematic review. *Front. Hum. Neurosci.*10.3389/fnhum.2024.1319520 (2024). 38371461 10.3389/fnhum.2024.1319520 PMC10869505

[7] Massano, J. & Garrett, C. Deep brain stimulation and cognitive decline in Parkinson’s disease: A clinical review. *Front. Neurol.*10.3389/fneur.2012.00066 (2012). 22557991 10.3389/fneur.2012.00066 PMC3337446

[8] Chen, J. W. et al. Electrode position and cognitive outcome following deep brain stimulation surgery. *J. Neurosurg.* **141**, 230–240 (2024). 38335523 10.3171/2023.11.JNS232164

[9] Olson, M. C., Shill, H., Ponce, F. & Aslam, S. Deep brain stimulation in PD: Risk of complications, morbidity, and hospitalizations: a systematic review. *Front. Aging Neurosci.* **15**, 1258190 (2023). 38046469 10.3389/fnagi.2023.1258190 PMC10690827

[10] Kleiner-Fisman, G. et al. Subthalamic nucleus deep brain stimulation: Summary and meta-analysis of outcomes. *Mov. Disord. Off. J. Mov. Disord. Soc.* **21**(Suppl 14), S290-304 (2006). 10.1002/mds.20962 16892449

[11] Rački, V. et al. Cognitive impact of deep brain stimulation in Parkinson’s disease patients: A systematic review. *Front. Hum. Neurosci.*10.3389/fnhum.2022.867055 (2022). 35634211 10.3389/fnhum.2022.867055 PMC9135964

[12] Cavallieri, F. et al. Predictors of long-term outcome of subthalamic stimulation in Parkinson disease. *Ann. Neurol.* **89**, 587–597 (2021). 33349939 10.1002/ana.25994

[13] Pal, G. D. et al. Cognitive effects of subthalamic nucleus deep brain stimulation in Parkinson’s disease with GBA1 pathogenic variants. *Mov. Disord.* **38**, 2155–2162 (2023). 37916476 10.1002/mds.29647 PMC10990226

[14] Kimmelman, J. et al. Risk of surgical delivery to deep nuclei: A meta-analysis. *Mov. Disord. Off. J. Mov. Disord. Soc.* **26**, 1415–1421 (2011). 10.1002/mds.23770 PMC4532377 21574186

[15] Whiting, A. C. et al. Peri-lead edema after deep brain stimulation surgery: A poorly understood but frequent complication. *World Neurosurg.* **124**, e340–e345 (2019). 30594699 10.1016/j.wneu.2018.12.092

[16] Nishiguchi, Y. et al. Relationship of brain edema after deep brain stimulation surgery with motor and cognitive function. *Heliyon* **8**, e08900 (2022). 35265762 10.1016/j.heliyon.2022.e08900 PMC8899698

[17] de Cuba, C. M. K. E. et al. Idiopathic delayed-onset edema surrounding deep brain stimulation leads: Insights from a case series and systematic literature review. *Parkinsonism Relat. Disord.* **32**, 108–115 (2016). 27622967 10.1016/j.parkreldis.2016.09.007

[18] Mira, R. G., Lira, M. & Cerpa, W. Traumatic brain injury: Mechanisms of glial response. *Front. Physiol.* **12**, 740939 (2021). 34744783 10.3389/fphys.2021.740939 PMC8569708

[19] Frank, A. et al. Serum neurofilament indicates that DBS surgery can cause neuronal damage whereas stimulation itself does not. *Sci. Rep.* **12**, 1446 (2022). 35087088 10.1038/s41598-022-05117-x PMC8795190

[20] Abdelhak, A. et al. Blood GFAP as an emerging biomarker in brain and spinal cord disorders. *Nat. Rev. Neurol.* **18**, 158–172 (2022). 35115728 10.1038/s41582-021-00616-3

[21] Axelsson, M. et al. Glial fibrillary acidic protein: A potential biomarker for progression in multiple sclerosis. *J. Neurol.* **258**, 882–888 (2011). 21197541 10.1007/s00415-010-5863-2

[22] Meier, S. et al. Serum glial fibrillary acidic protein compared with neurofilament light chain as a biomarker for disease progression in multiple sclerosis. *JAMA Neurol.* **80**, 287–297 (2023). 36745446 10.1001/jamaneurol.2022.5250 PMC10011932

[23] Pilotto, A. et al. Plasma NfL, GFAP, amyloid, and p-tau species as Prognostic biomarkers in Parkinson’s disease. *J. Neurol.* **271**, 7537–7546 (2024). 39249107 10.1007/s00415-024-12669-7 PMC11588809

[24] Tang, Y. et al. Plasma GFAP in Parkinson’s disease with cognitive impairment and its potential to predict conversion to dementia. *Npj Park. Dis.* **9**, 1–5 (2023). 10.1038/s41531-023-00447-7 PMC9911758 36759508

[25] Che, N. et al. Plasma GFAP as a prognostic biomarker of motor subtype in early Parkinson’s disease. *Npj Park. Dis.* **10**, 48 (2024). 10.1038/s41531-024-00664-8 PMC10907600 38429295

[26] Liu, X. et al. Utility of serum neurofilament light chain and glial fibrillary acidic protein as diagnostic biomarkers of freezing of gait in Parkinson’s disease. *Brain Res.* **1822**, 148660 (2024). 37924925 10.1016/j.brainres.2023.148660

[27] You, H. et al. Evaluation of blood glial fibrillary acidic protein as a potential marker in Huntington’s disease. *Front. Neurol.*10.3389/fneur.2021.779890 (2021). 34867769 10.3389/fneur.2021.779890 PMC8639701

[28] Castaño-Leon, A. M. et al. Serum assessment of traumatic axonal injury: The correlation of GFAP, t-Tau, UCH-L1, and NfL levels with diffusion tensor imaging metrics and its prognosis utility. *J. Neurosurg.* **138**, 454–464 (2022). 35901687 10.3171/2022.5.JNS22638

[29] Ferrari, F. et al. Quantification and prospective evaluation of serum NfL and GFAP as blood-derived biomarkers of outcome in acute ischemic stroke patients. *J. Cereb. Blood Flow Metab.* **43**, 1601–1611 (2023). 37113060 10.1177/0271678X231172520 PMC10414005

[30] Yalachkov, Y. et al. Effect of estimated blood volume and body mass index on GFAP and NfL levels in the serum and CSF of patients with multiple sclerosis. *Neurol. Neuroimmunol. Neuroinflam.* **10**, e200045 (2023). 10.1212/NXI.0000000000200045 PMC9673750 36316116

[31] Wang, X., Shi, Z., Qiu, Y., Sun, D. & Zhou, H. Peripheral GFAP and NfL as early biomarkers for dementia: Longitudinal insights from the UK Biobank. *BMC Med.* **22**, 192 (2024). 38735950 10.1186/s12916-024-03418-8 PMC11089788

[32] Ma, L.-Z. et al. Serum neurofilament dynamics predicts cognitive progression in de novo Parkinson’s disease. *J. Park. Dis.* **11**, 1117–1127 (2021). 10.3233/JPD-212535 33935105

[33] Ma, W. et al. Elevated levels of serum neurofilament light chain associated with cognitive impairment in vascular dementia. *Dis. Markers* **2020**, 6612871 (2020). 33204362 10.1155/2020/6612871 PMC7652600

[34] Azizi, S. et al. A kinetic model for blood biomarker levels after mild traumatic brain injury. *Front. Neurol.* **12**, 668606 (2021). 34295300 10.3389/fneur.2021.668606 PMC8289906

[35] Khalil, M. et al. Serum neurofilament light levels in normal aging and their association with morphologic brain changes. *Nat. Commun.* **11**, 812 (2020). 32041951 10.1038/s41467-020-14612-6 PMC7010701

[36] Manouchehrinia, A. et al. Confounding effect of blood volume and body mass index on blood neurofilament light chain levels. *Ann. Clin. Transl. Neurol.* **7**, 139–143 (2020). 31893563 10.1002/acn3.50972 PMC6952306

[37] Frank, A. et al. Serum neurofilament indicates accelerated neurodegeneration and predicts mortality in late-stage Parkinson’s disease. *Npj Park. Dis.* **10**, 14 (2024). 10.1038/s41531-023-00605-x PMC10776839 38195715

[38] Schnalke, N. et al. Morbidity milestones demonstrate long disability-free survival in Parkinson’s disease patients with deep brain stimulation of the subthalamic nucleus. *Mov. Disord. Clin. Pract.* **10**, 569–578 (2023). 37070057 10.1002/mdc3.13698 PMC10105113

[39] Kempster, P. A., O’Sullivan, S. S., Holton, J. L., Revesz, T. & Lees, A. J. Relationships between age and late progression of Parkinson’s disease: A clinico-pathological study. *Brain* **133**, 1755–1762 (2010). 20371510 10.1093/brain/awq059

[40] Borellini, L. et al. Peri-lead edema after deep brain stimulation surgery for Parkinson’s disease: A prospective magnetic resonance imaging study. *Eur. J. Neurol.* **26**, 533–539 (2019). 30358915 10.1111/ene.13852

[41] Li, D. et al. Neurochemical regulation of the expression and function of glial fibrillary acidic protein in astrocytes. *Glia* **68**, 878–897 (2020). 31626364 10.1002/glia.23734

[42] Kouli, A. et al. Neuroinflammation is linked to dementia risk in Parkinson’s disease. *Brain* **147**, 923–935 (2023). 10.1093/brain/awad322 PMC10907093 37757857

[43] Defer, G.-L., Widner, H., Marié, R.-M., Rémy, P. & Levivier, M. Core assessment program for surgical interventional therapies in Parkinson’s disease (CAPSIT-PD). *Mov. Disord.* **14**, 572–584 (1999). 10435493 10.1002/1531-8257(199907)14:4<572::aid-mds1005>3.0.co;2-c

[44] Foley, J. A., Foltynie, T., Limousin, P. & Cipolotti, L. Standardised neuropsychological assessment for the selection of patients undergoing DBS for Parkinson’s disease. *Park. Dis.* **2018**, 4328371 (2018). 10.1155/2018/4328371 PMC6009029 29971141

[45] Graham, N. S. N. et al. Axonal marker neurofilament light predicts long-term outcomes and progressive neurodegeneration after traumatic brain injury. *Sci. Transl. Med.* **13**, eabg9922 (2021). 34586833 10.1126/scitranslmed.abg9922

[46] Shahim, P. et al. Neurofilament light as a biomarker in traumatic brain injury. *Neurology* **95**, e610–e622 (2020). 32641538 10.1212/WNL.0000000000009983 PMC7455357

[47] García-Pérez, M. A. Use and misuse of corrections for multiple testing. *Methods Psychol.* **8**, 100120 (2023).

