# Supplementary material for: Serum glial fibrillary protein reflects early brain injury dynamics and cognitive changes after deep brain stimulation surgery
Source: Sci Rep. 2025 May 13;15:16537. doi: 10.1038/s41598-025-00399-3 (PMC12075788; doi:10.1038/s41598-025-00399-3)
Supplement: Supplementary file 1 — Supplementary Information. [file 41598_2025_399_MOESM1_ESM.docx]

**Supplementary Figures**


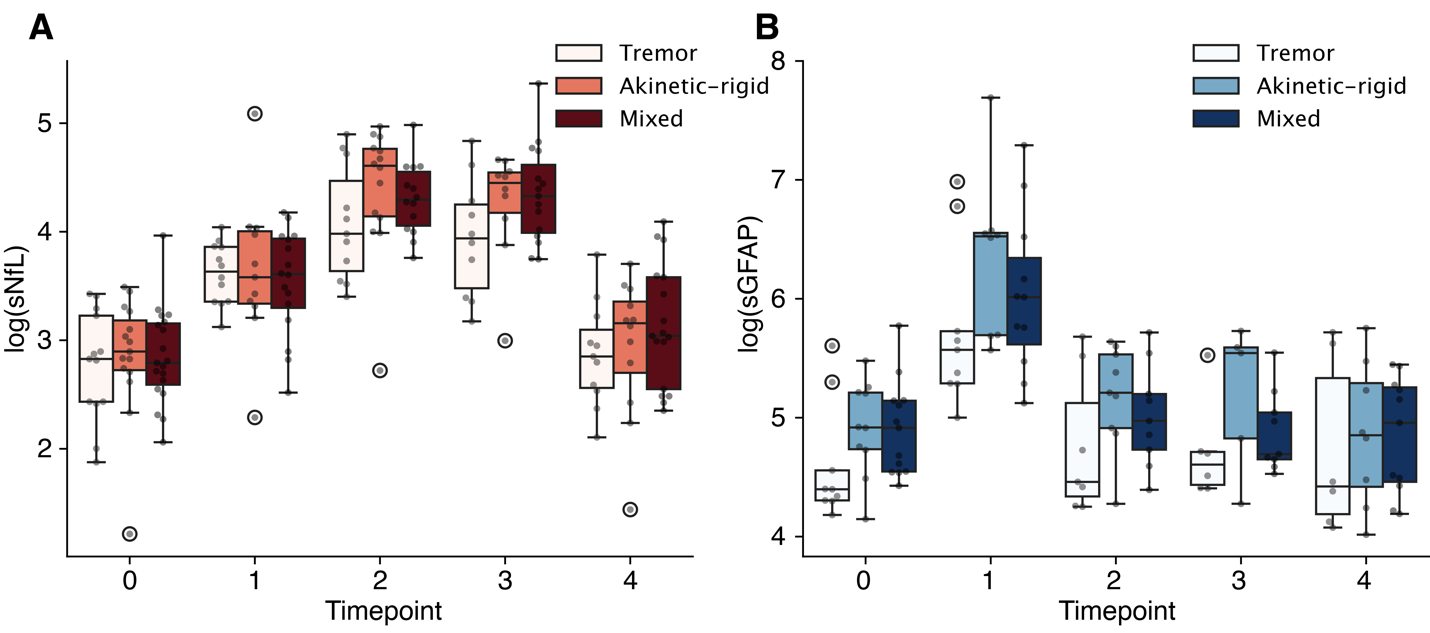


**Supplementary Figure 1.** Dynamics of log(sNfL) (A) and log(sGFAP) (B) for different disease phenotypes of PD patients. Circled dots show outliers (>75^th^ percentile + 1.5 Interquartile Range or <25^th^ percentile - 1.5 Interquartile Range). A mixed linear model analysis of log(sNfL) showed a significant effect of timepoint (with significantly higher log(sNfL) values on timepoints 1,2 and 3 compared to baseline, p < 0.001 for all) and a significant interaction term for disease phenotype (Equivalent vs Tremor) with timepoint 3 (p = 0.030). The mixed linear model analysis of log(sGFAP) showed a significant effect of timepoint (with significantly higher log(sGFAP) values on timepoints 1 compared to baseline, p < 0.001) and a significant interaction term for disease phenotype (Akinetic-rigit vs Tremor) with timepoint 1 (p = 0.034). For both models the main effect of disease phenotype was not significant.
